# Current Knowledge and Future Perspectives of Buttock Augmentation: A Bibliometric Analysis from 1999 to 2021

**DOI:** 10.1007/s00266-022-03140-x

**Published:** 2022-10-25

**Authors:** Yuxuan Dai, Yu Chen, Yiming Hu, Lianbo Zhang

**Affiliations:** 1 Department of Plastic Surgery, China-Japan Union Hospital of Jilin University, 126 Xiantai street, Erdao District, Changchun, 130000 Jilin Province People’s Republic of China; 2 Division of Thyroid Surgery, Sun Yat-sen Memorial Hospital, Sun Yat-sen University, Guangzhou, 510120 China; 3 Department of Plastic and Aesthetic Surgery, The Second Xiangya Hospital of Central South University, Changsha, 410011 China

**Keywords:** Buttock augmentation, Buttock aesthetics, Autologous fat grafting, Buttock implant, Gluteoplasty, Bibliometric analysis

## Abstract

**Background:**

The number of patients undergoing buttock augmentation surgery has increased rapidly with time, changes in people’s aesthetic perceptions, and the increased concern for their shape. The number of publications regarding buttock augmentation has also continued to increase. However, no bibliometric analysis concerning buttock augmentation has been published. This study aimed to provide a qualitative and quantitative evaluation of buttock augmentation-related publications using bibliometric analysis and information on research hotspots and trends in this field.

**Methods:**

The buttock augmentation-related publications published between 1999 and 2021 were extracted from the Web of Science Core Collection (WOSCC) database for analysis. The data were analysed and presented using VOSviewer and Microsoft Excel.

**Results:**

There were 492 articles in the (WOSCC) database, including 442 (89.84%) original research articles, with the number of publications increasing each year. The USA (208 publications, 42.28%) is the leading contributor in this field and has a high academic reputation. The most productive and co-cited journal on this subject is “Plastic and Reconstructive Surgery” (66 publications, 13.41%, 2200 citations). Cardenas-Camarena (9 publications, 1.83%, 158 citations) was the most published and co-cited author. Research hotspots include the following three topics: experience and technology of buttock augmentation, autologous fat buttock augmentation and its safety, and buttock aesthetics study. There will be more publications in the future, and research trends will focus on silicone implants, safety, satisfaction, and autologous fat grafting.

**Conclusion:**

Buttock augmentation research is rapidly evolving, and this study provides a perspective view of buttock augmentation research in Plastic and Reconstructive Surgery.

**Level of Evidence III:**

This journal requires that authors assign a level of evidence to each article. For a full description of these Evidence-Based Medicine ratings, please refer to the Table of Contents or the online Instructions to Authors www.springer.com/00266.

## Introduction

As a crucial component to undertake the aesthetic unit of the waist and legs, the buttocks are receiving increasing attention from plastic surgeons and patients. According to the annual statistics of the American Society of Plastic Surgery (FY 2019), buttock augmentation has increased by 90.3%, ranking 6th in the number of all plastic surgery procedures [1]. In 1969, Bartles et al. [2] were the first to place breast silicone implants into the buttocks for idiopathic unilateral gluteal muscle atrophy. In 1973, Cocke and Rickeson performed the first successful buttock augmentation for cosmetic purposes using a round gluteal silicone implant [3]. Although buttock augmentation with prosthesis originated in the USA, it did not become popular subsequently.

With the increase in buttock augmentation surgery in recent years, a consensus on the aesthetic understanding and aesthetic standards of the buttocks has gradually formed, and plastic surgeons have created aesthetic measures such as the golden waist-to-hip ratio and the four depressions (sacral fossa, gluteal groove, subgluteal crease, and lateral buttock depression) based on their proportions. The existing methods of buttock augmentation include buttock augmentation with implants, buttock augmentation with autologous fat grafting, buttock augmentation with hyaluronic acid fillers, buttock augmentation with autologous tissue flaps, and combined surgical approaches. Among these, implants and autologous fat fillers are the most common surgical approaches [4].

A bibliometric analysis is a qualitative and quantitative examination of published publications such as academic articles or books that uses a combination of mathematical and statistical methodologies to assess scientific output, research hotspots, and research trends in a field [5]. With the increase in buttock augmentation procedures in recent years, the number of publications on buttock augmentation-related research has also increased. However, no bibliometric analysis of buttock augmentation-related research has been published to date. Hence, the current study performed a bibliometric analysis to evaluate the field to provide the current status of development, research hotspots, and emerging trends in buttock augmentation, as well as to predict future research objectives.

## Materials and Methods

### Search Strategy

Publications were downloaded from the Web of Science Core Collection (WOSCC) database using the following search terms: TS = (gluteoplasty) OR (gluteal myoplasty) OR (buttock myoplasty) OR (gluteal augmentation) OR (buttock augmentation) OR (gluteal implant) OR (buttock implant) OR (gluteal lipoaugmentation) OR (buttock lipoaugmentation) OR (buttock fat grafting) OR (gluteal fat grafting). On July 15, 2022, the database was searched, and the publications included in the analysis comprised only original research articles and reviews published in English. The search indicated that the first article on buttock augmentation in WOSCC was published in 1999. Hence, we used 1999 as the starting point of the search year and 2021 as the end point of the search year and analysed all publications.

### Data Collection and Analysis

Two researchers independently selected and collected the literature and cross-checked references, and any conflicts were resolved by the senior researcher after reading the full text for inclusion in the study. Study data included: country/region, institution, author, journal, keywords, number of annual publications, and number of publications by the authors. The data were downloaded in plain text format. The h-index is a new method for assessing academic achievement. The h-index can precisely reflect one’s academic achievement. The “impact factor (IF)” of the journals and “Quartile” in the category were also obtained and utilized to estimate the scientific impact of the country/region. The obtained data are presented in tables and figures using Microsoft Excel 2016.

### Visual Analytics

VOSviewer (1.6.18), a free JAVA-based software, was developed in 2009 by Van Eck and Waltman of The Centre for Science and Technology Studies (CWTS), Leiden University, The Netherlands. Compared to other bibliometric software, the best application of VOSviewer is its graphical presentation capability, adaptability for large-scale data, and versatility in adapting to multiple databases with varied formats of source data [6, 7]. The core concept of VOSviewer software design is “co-occurrence clustering”, which means that two things appearing at the same time are related to each other; there are multiple types of such related relationships with different strengths and directions; clustering is based on measures of relationship strengths, and directions allow for the identification of different types of groups. Circles and labels form an element, and the size of the element depends on the size of the node, the strength of the link, the number of citations, etc. The colour of the element represents the cluster it belongs to, different clusters are represented by different colours, and each cluster can be seen through this view. Total link strength (TLS) indicates the degree of cooperation between an item and other items, and its value is proportional to the level of cooperation.

## Results

**Fig. 1:**
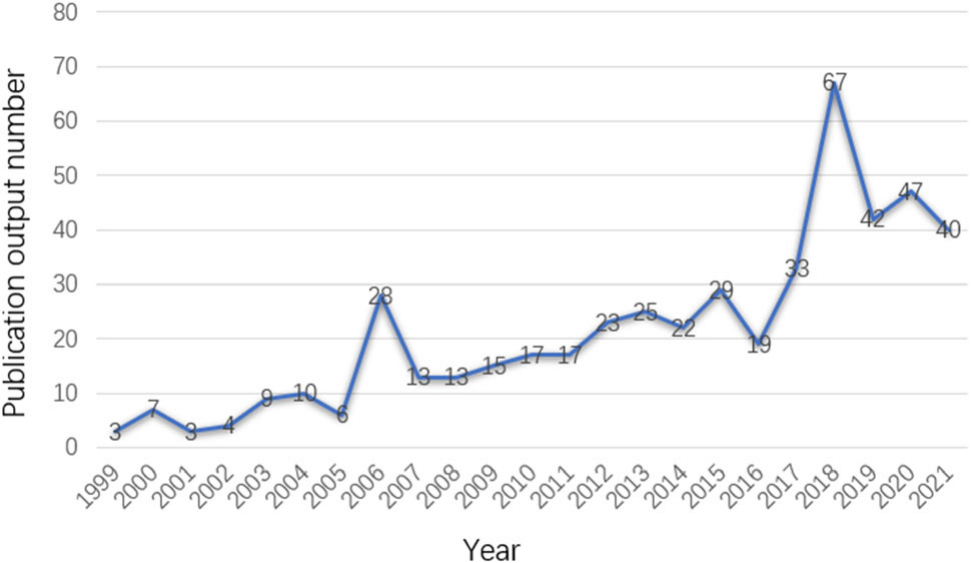
Annual worldwide publication output

A total of 492 publications were identified between 1999 to 2021 from the WOSCC online database, including 442 (89.84%) original research articles and 50 (10.16%) review articles. The variation in the number of publications each year is depicted in Fig. 1. The maximum number of publications was 67 (13.62%) in 2018, with an overall upward trend in the number of annual publications. A total of 143 (29.07%) were open access.

### Analysis of Countries/Regions

**Fig. 2:**
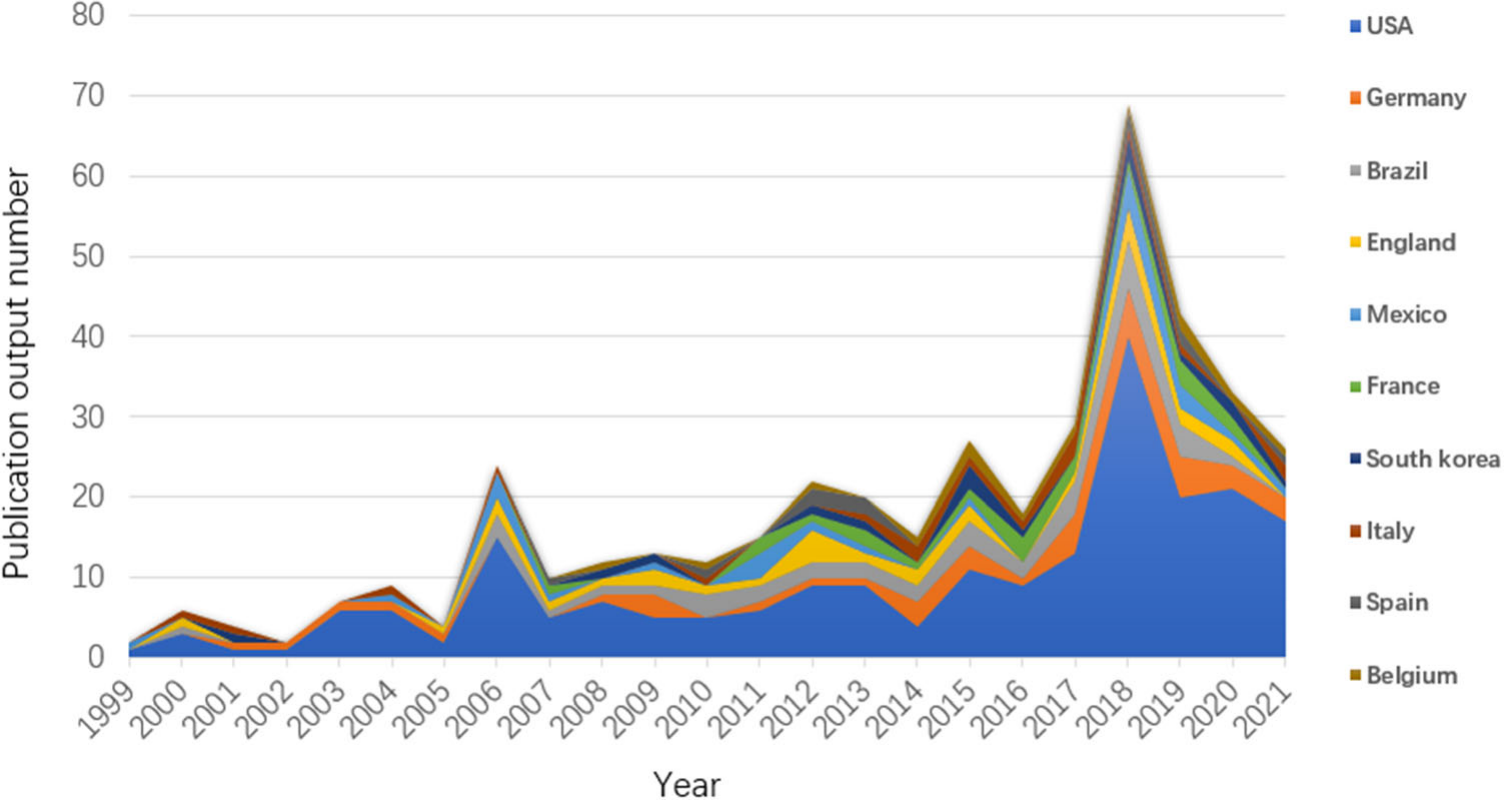
Annual national publication output of 10 most productive countries/regions

**Fig. 3:**
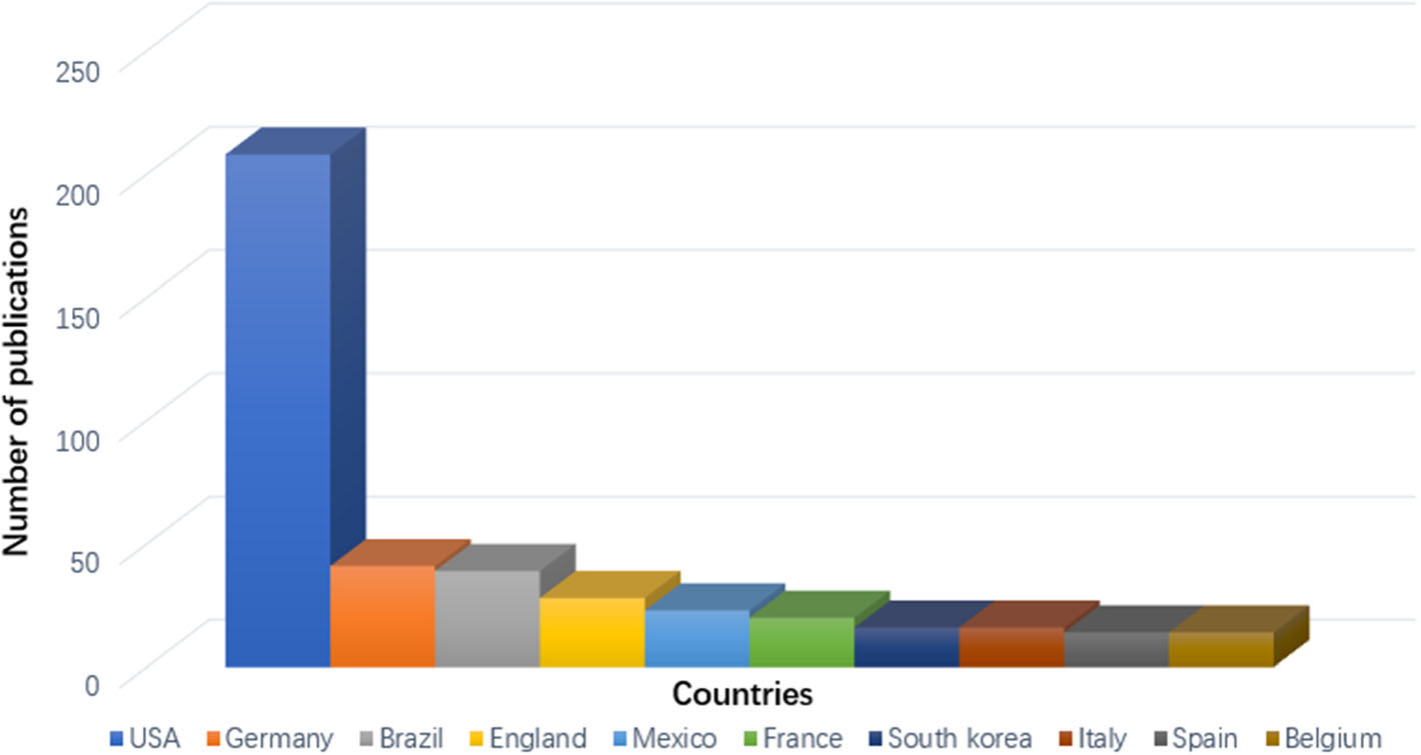
Top 10 countries/regions according to total number of publications on buttock augmentation

**Fig. 4:**
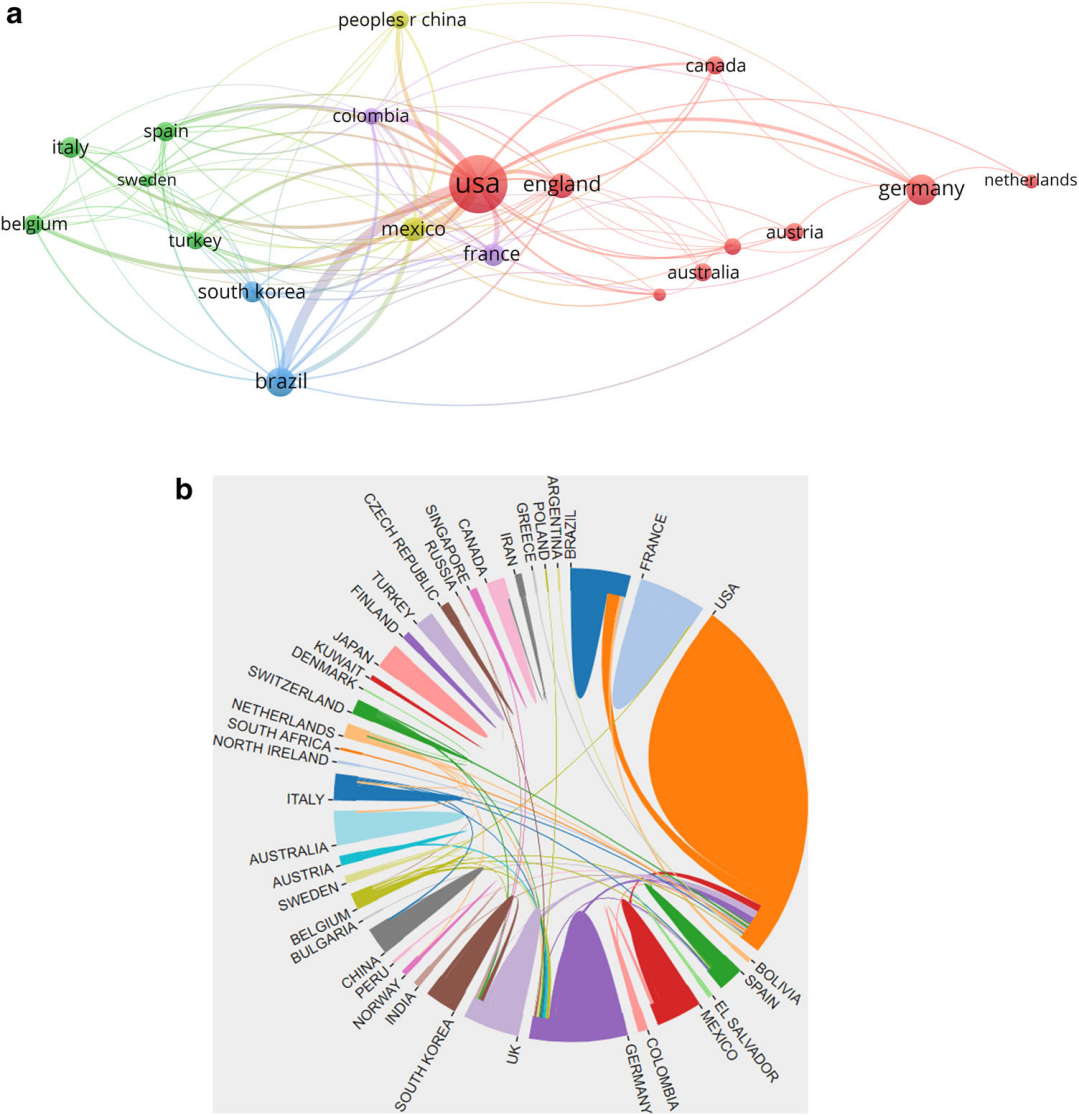
(**A**, **B**) Network visualization map of countries/regions related to buttock augmentation research

The publications were distributed in 55 countries/regions. Figure 2 depicts the annual growth trend of buttock augmentation publications of the top 10 countries/regions. The top 10 countries/regions with the most publications are shown in Fig. 3, with the USA producing the highest number of buttock augmentation publications than any other country. Table 1 displays the extensive data about the top 10 countries/regions with the most publications. The USA had the highest number of relevant publications (208, 42.28%), followed by Germany (41, 8.33%), Brazil (39, 7.93%), England (28, 5.69%), and Mexico (23, 4.67%). Figure 4 depicts the network visualization map of countries/regions involved in buttock augmentation research, with the size of the circular nodes representing the number of publications published by the corresponding countries and the connecting lines between the nodes representing the collaboration relationship between countries/regions. The USA has the most number of publications (208), the most collaborating countries (19), TLS (713), and total citations (4015), suggesting that the USA is a major contributor to buttocks research and has a high academic reputation. The USA has far more publications in this field than any other country, five times more than Germany (the second highest number of publications). Moreover, Italy has the highest number of citations per publication (CPP: 29.94).

**Table 1:** Top 10 productive countries/regions of buttock augmentation research

Rank	Country/region	Records	Percentage (%)	Citations	Citations per publication	Total link strength (TLS)
1	USA	208	42.28	4015	19.30	713
2	Germany	41	8.33	740	18.05	40
3	Brazil	39	7.93	505	12.95	321
4	England	28	5.69	608	21.71	67
5	Mexico	23	4.67	503	21.87	311
6	France	20	4.07	275	13.75	90
7	South korea	16	3.25	83	5.19	48
8	Italy	16	3.25	479	29.94	33
9	Spain	14	2.85	160	11.43	55
10	Belgium	14	2.85	314	22.43	52

### Analysis of Institutions

All publications were distributed among 778 institutions. The top 10 institutions with the most publications are listed in Table 2. The leading institution was New York University (60 publications, 12.20%), followed by Louisiana State University (50 publications, 10.16%), University Estado Rio De Janeiro (40 publications, 8.13%), Rutgers New Jersey Medical School (35 publications, 7.11%), and Ohio State University (35 publications, 7.11%). Most of the 10 institutions with the highest output of buttock augmentation-related publications were from the USA, indicating the strong academic influence of the USA in this field. Louisiana State University (USA) had the highest number of citations per publication (9.14), indicating that this institution publishes a large number of high-quality publications and is a major contributor to the development of the field.

**Table 2:** Top 10 productive institutions of buttock augmentation research

Rank	Institution	Records	Percentage (%)	Citations	Citations per publication	TLS
1	New York University	60	12.20	261	4.35	28
2	Louisiana State University	50	10.16	457	9.14	24
3	University Estado Rio De Janeiro	40	8.13	109	2.73	24
4	Rutgers New Jersey Medical School	35	7.11	74	2.11	30
5	Ohio State University	35	7.11	61	1.74	22
6	Mayo clinic	35	7.11	81	2.31	15
7	University of Texas Southwestern Medical Center, Dallas	30	6.10	127	4.23	49
8	University of Sao Paulo	30	6.10	59	1.97	31
9	Stanford University	30	6.10	74	2.47	9
10	The Catholic University of Korea	30	6.10	32	1.07	6

**Fig. 5:**
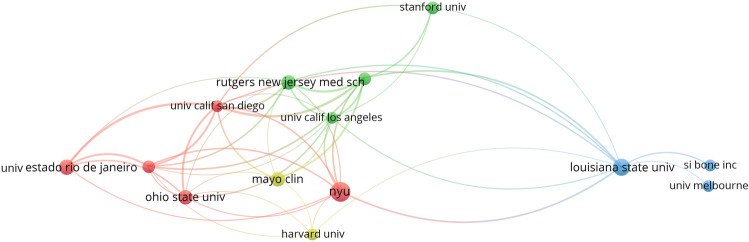
Network visualization map of institutions related to buttock augmentation research

The visualization of the network of collaborating institutions based on the number of citations is depicted in Fig. 5, with a minimum unit of measurement of five for each institution’s publications and a total of 14 nodes, with the lines between the nodes representing collaborative relationships, divided into four clusters. Figure 5 shows that the leading institutions collaborating with New York University are Louisiana State University, Rutgers New Jersey Medical School, University of Texas Southwestern Medical Center, Dallas, University of Sao Paulo, and University of California San Diego.

### Analysis of Author and Co-cited Authors

**Fig. 6:**
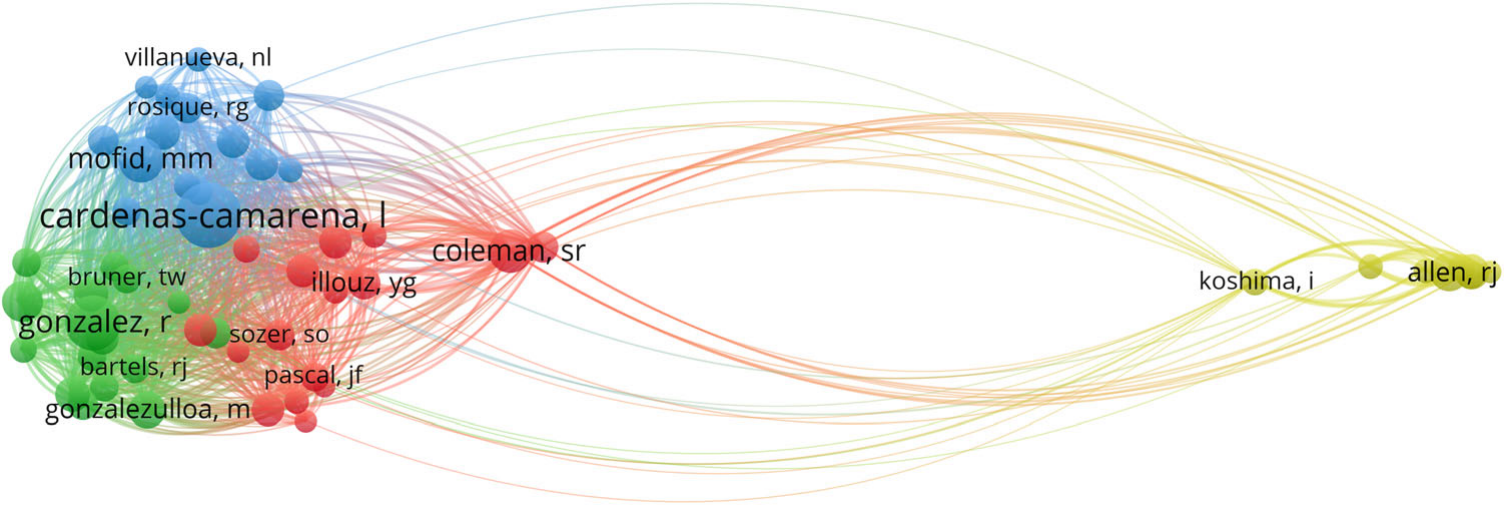
Network visualization map of co-cited authors related to buttock augmentation research

A total of 2058 authors have published articles on the augmentation of the buttocks. The top 10 authors with the most publications and co-cited authors are listed in Table 3. Cardenas-Camarena (9 publications, 1.83%) published the most papers, followed by Aboudib JH (7 publications, 1.42%) and Serra F (6 publications, 1.22%). The top three co-cited authors were Cardenas-Camarena (158 citations, TLS: 1993), Gonzalez R (118 citations, TLS: 1398), and Mofid MM (158 citations, TLS: 821). Cardenas-Camarena is the most published and co-cited author in buttock augmentation-related publications, indicating his high academic achievement in this field. Figure 6 depicts the network visualization of co-cited authors, using a minimum co-citation count of 20 as the unit of measurement. A total of 56 authors satisfied the condition, and the analysis results showed four clusters, with the largest nodes representing the most co-cited, namely Coleman SR (cluster 1, red areas, 16 items), Gonzalez R (cluster 2, green areas, 16 items), Cardenas-Camarena (cluster 3, blue areas, 15 items), and Allen RJ (cluster 4, yellow areas, 8 items).

**Table 3:** Top 10 productive authors and co-cited authors

Rank	Author	Records	Percentage (%)	Rank	Co-cited author	Citation	TLS
1	Cardenas-Camarena	9	1.83	1	Cardenas-Camarena	158	1993
2	Aboudib JH	7	1.42	2	Gonzalez R	118	1398
3	Serra F	6	1.22	3	Mofid MM	75	821
4	Senderoff DM	5	1.02	4	Coleman SR	70	634
5	Mendieta CG	5	1.02	5	Serra F	64	823
6	Gonzalez R	5	1.02	6	Allen RJ	59	369
7	Centeno RF	5	1.02	7	Gonzalezulloa M	51	618
8	Villanueva Nl	5	1.02	8	Mendieta CG	49	729
9	Cher D	5	1.02	9	Sinno S	49	584
10	Liu LQ	5	1.02	10	Vergara R	48	559

### Analysis of Journals and Co-cited Journals

**Fig. 7:**
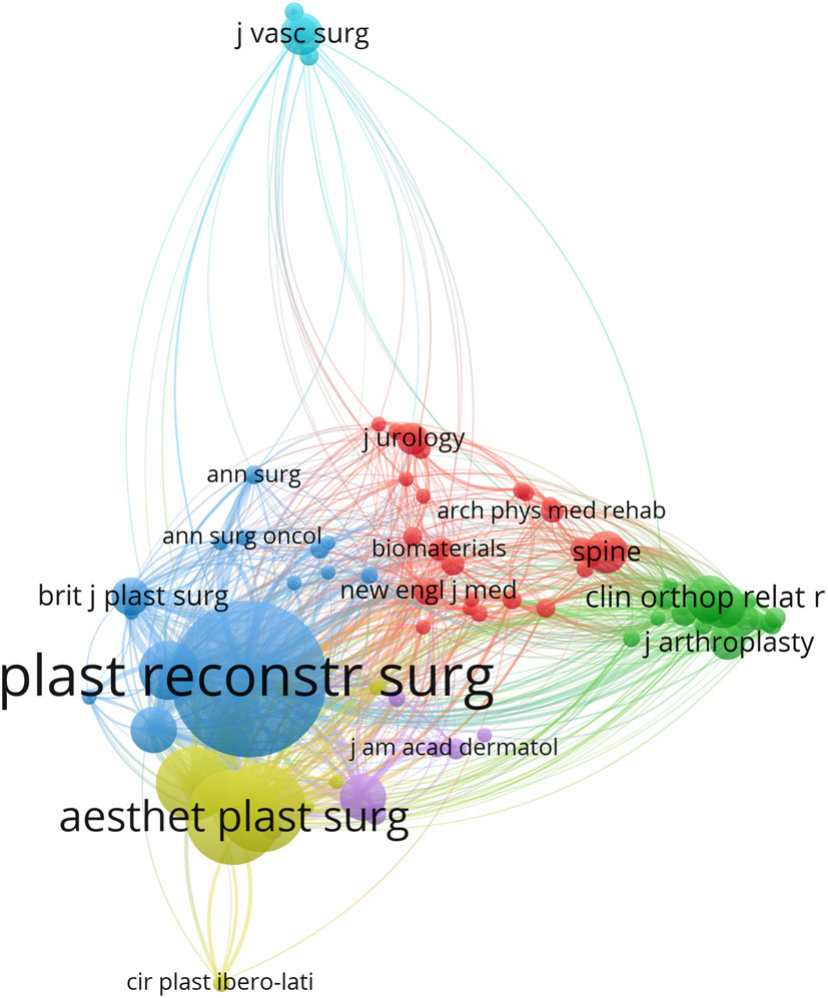
Network visualization map of co-cited journals related to buttock augmentation research

A total of 206 journals published on buttock augmentation. Tables 4 and 5 show the top 10 most published and most co-cited articles on buttock augmentation. Plastic and Reconstructive Surgery (66 publications, 13.41%) published the most publications in this field, followed by Aesthetic Surgery Journal (38 publications, 7.72%) and Aesthetic Plastic Surgery (38 publications, 7.72%). The top three most co-cited journals were Plastic and Reconstructive Surgery (2200 citations, TLS: 39,999), Aesthetic Plastic Surgery (831 citations, TLS: 16,880), and Aesthetic Surgery Journal (716 citations, TLS: 15,868). Plastic and Reconstructive Surgery is the most productive and co-cited journal in this field, indicating that this journal can serve as a quality information resource for researchers studying the augmentation of buttocks. The network visualization of co-cited journals is shown in Fig. 7.

**Table 4:** Top 10 productive journals of buttock augmentation research

Rank	Journal	Records	Percentage (%)	IF(%,2021-2022)	H-Index	Citations per publication	Quartile in category
1	Plastic and Reconstructive Surgery	66	13.41	5.169	160	29.17	Q1
2	Aesthetic Surgery Journal	38	7.72	4.485	48	13.66	Q1
3	Aesthetic Plastic Surgery	38	7.72	2.708	59	13.68	Q3
4	Clinics in Plastic Surgery	30	6.10	2.53	57	16.93	Q3
5	Annals of Plastic Surgery	24	4.88	1.763	83	14.54	Q4
6	Plastic and Reconstructive Surgery-global Open	12	2.44		18	6.08	
7	Journal of Plastic Reconstructive and Aesthetic Surgery	9	1.83	3.022	82	12.44	Q2
8	Annals of Vascular Surgery	6	1.22	1.607	67	7.00	Q4
9	Dermatologic Surgery	5	1.02	2.914	113	15.20	Q2
10	Skeletal Radiology	5	1.02	2.128	81	3.40	Q3

**Table 5:** Top 10 most frequently co-cited journals of buttock augmentation research

Rank	Journal	Citations	TLS	IF(%,2021-2022)	H-Index	Quartile in category
1	Plastic and Reconstructive Surgery	2200	39999	5.169	160	Q1
2	Aesthetic Plastic Surgery	831	16880	2.708	59	Q3
3	Aesthetic Surgery Journal	716	15868	4.485	48	Q1
4	Clinics in Plastic Surgery	504	12588	2.53	57	Q3
5	Annals of Plastic Surgery	312	9947	1.763	83	Q4
6	Journal of Plastic Reconstructive and Aesthetic Surgery	197	6184	3.022	82	Q2
7	Dermatologic Surgery	193	3987	2.914	113	Q2
8	Clinical Orthopaedics and Related Research	192	3937	4.755	185	Q1
9	Journal of Bone and Joint Surgery-American Volume	186	3782	6.558	235	Q1
10	Journal of Vascular Surgery	160	1624	4.860	178	Q1

### Analysis of References

**Fig. 8:**
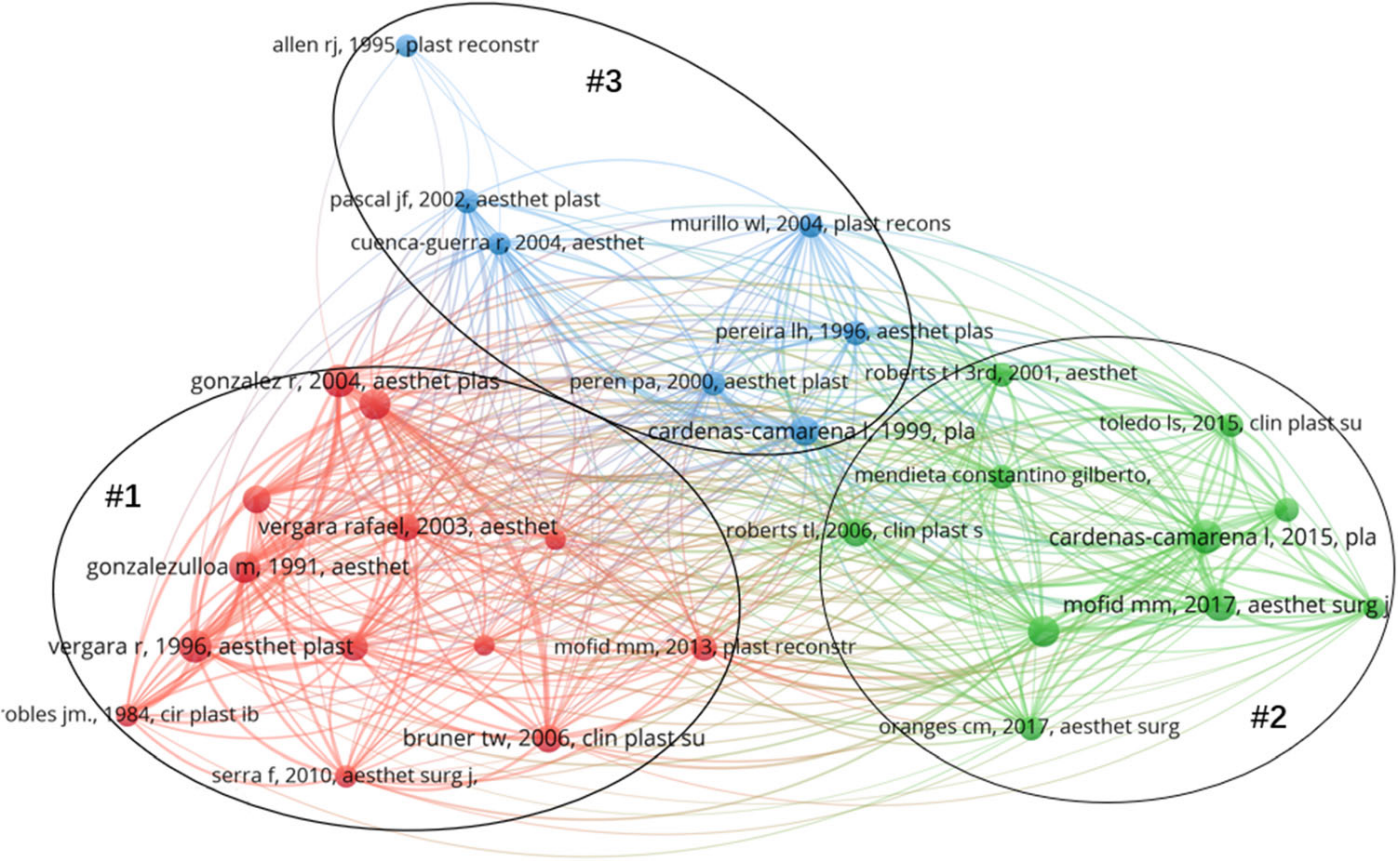
Network visualization map of co-cited references related to buttock augmentation research

A visual network map of co-cited references in this field with 30 nodes, three different coloured clusters, and 2783 links is depicted in Fig. 8. The size of the nodes indicates the number of citations of the publication. Table 6 summarizes the top 10 most co-cited articles based on the title, author, journal, impact factor, number of citations, year of publication, and Quartile in category. According to the top 10 most co-cited references, the article “Intramuscular gluteal implants” published in Aesthetic Plastic Surgery (IF: 2.708), was the most co-cited article. The article is authored by Vergara R et al. and co-cited 46 times. The next most co-cited articles were “Gonzalez et al., 2004, Aesthet Plast Surg”, “Mofid et al. “Cardenas-Camarena et al., 2015, Plast Reconstr Sur”, and “Gonzalezulloa et al., 1991, Aesthet Plast Surg”. More than half of the articles were published in Q1 (Quartile in category). There are three clusters, and an evaluation of the leading publications in each cluster reveals that they correspond to three research hotspots: experience and technology of buttock augmentation (Cluster 1, red area), autologous fat buttock augmentation and its safety (Cluster 2, green area), and buttock aesthetics study (Cluster 3, blue area) (Fig. 8).

**Table 6:** Top 10 most frequently co-cited references of buttock augmentation

Rank	Title	Journal IF (2021-2022)	Author	Publication time	co-citations	Quartile in category
1	Intramuscular gluteal implants	Aesthetic Plastic Surgery IF(2.708)	Vergara R	1996	46	Q2
2	Augmentation gluteoplasty: the XYZ method	Aesthetic Plastic Surgery IF(2.708)	Gonzalez R	2004	45	Q2
3	Report on Mortality from Gluteal Fat Grafting: Recommendations from the ASERF Task Force	Aesthetic Surgery Journal IF(4.485)	Mofid MM	2017	45	Q1
4	Deaths Caused by Gluteal Lipoinjection: What Are We Doing Wrong?	Plastic and Reconstructive Surgery IF(5.169)	Cardenas-Camarena l	2015	44	Q1
5	Gluteoplasty: a ten-year report	Aesthetic Plastic Surgery IF(2.708)	Gonzalezulloa M	1991	43	Q2
6	Determining the Safety and Efficacy of Gluteal Augmentation: A Systematic Review of Outcomes and Complications	Plastic and Reconstructive Surgery IF(5.169)	Sinno S	2016	43	Q1
7	Gluteoplasty	Aesthetic Surgery Journal IF(4.485)	Mendieta Constantino G	2003	40	Q1
8	Combined gluteoplasty: liposuction and lipoinjection	Plastic and Reconstructive Surgery IF(5.169)	Cardenas-Camarena l	1999	38	Q1
9	Intramuscular gluteal implants: 15 years of experience	Aesthetic Surgery Journal IF(4.485)	Vergara R	2003	34	Q1
10	Subfascial technique for gluteal augmentation	Aesthetic Surgery Journal IF(4.485)	De La Pena Jose Abel	2004	33	Q1

### Analysis of Keyword Co-occurrence Clusters

**Fig. 9:**
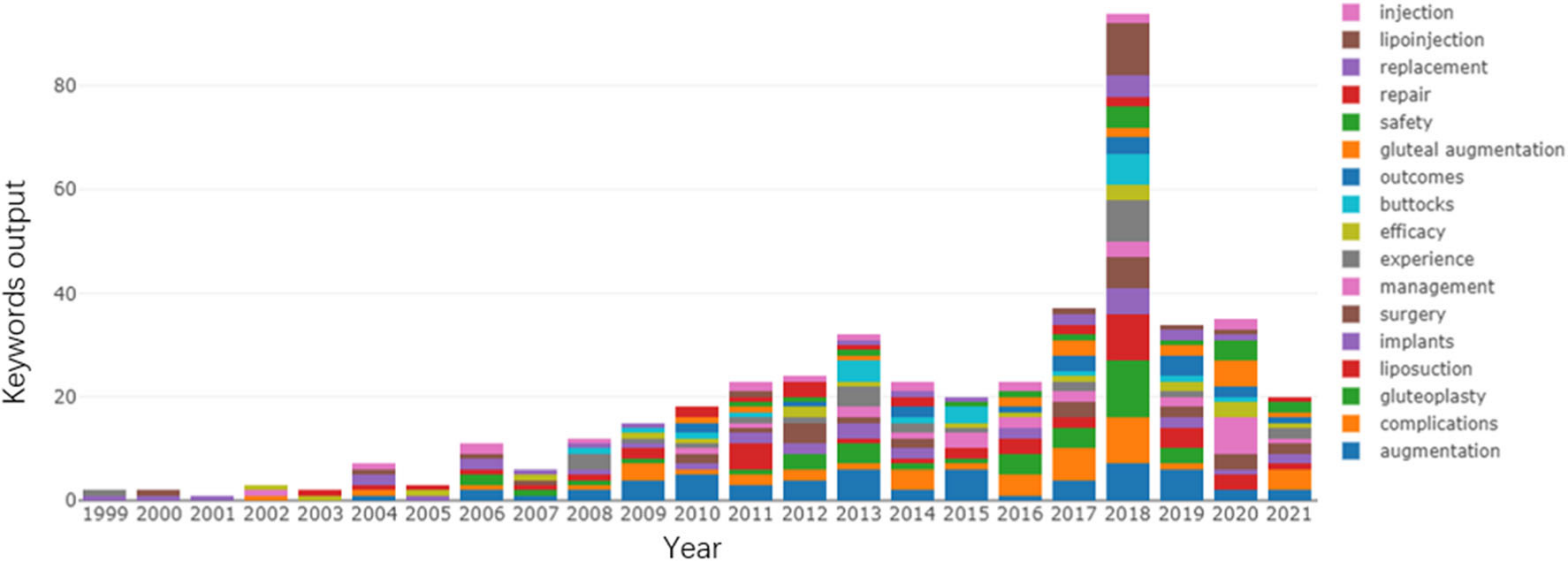
Annual keywords publication output of the buttock augmentation research

**Fig. 10:**
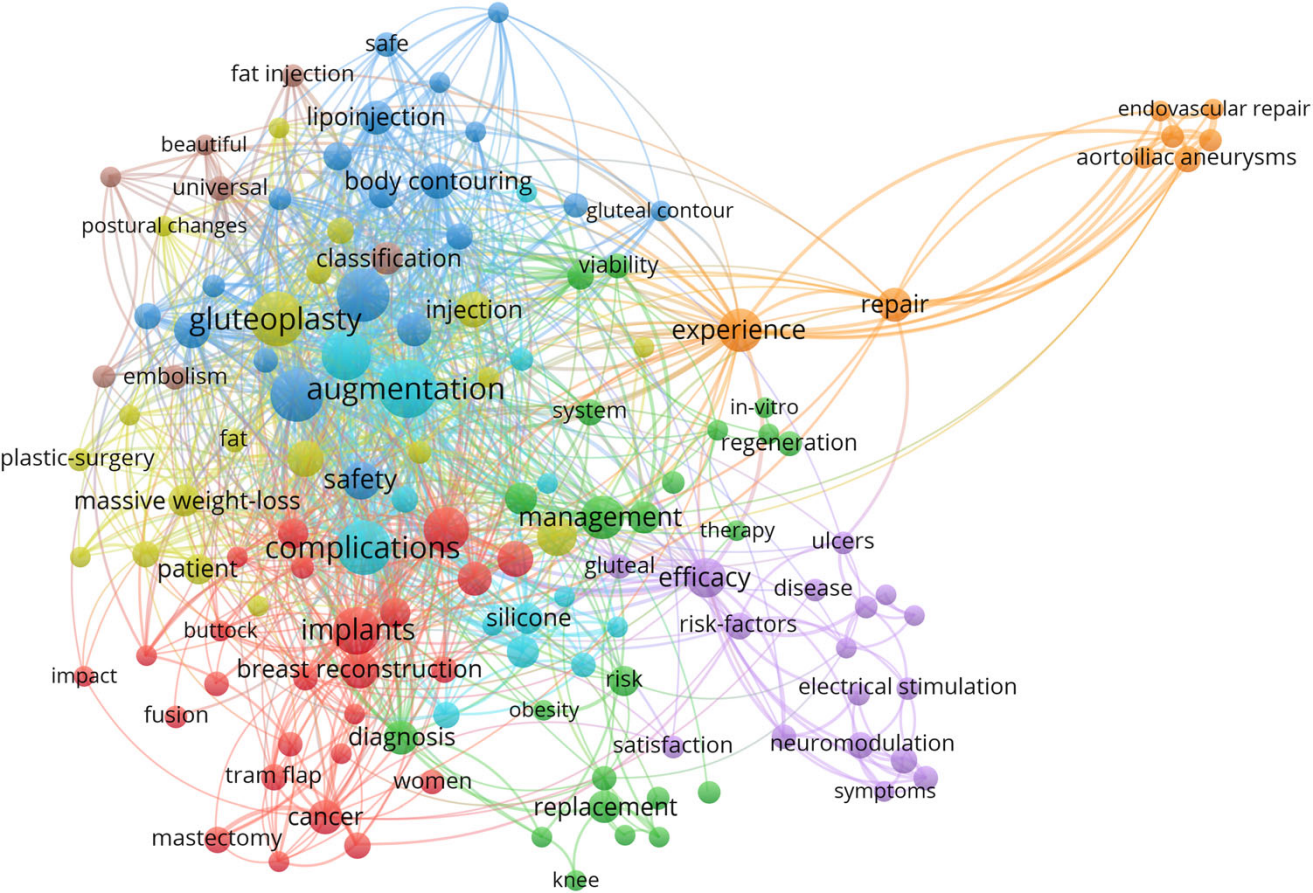
Network visualization map of keywords co-occurrence related to buttock

A total of 2252 keywords were mentioned in 492 publications. The annual keywords publication output of the buttock augmentation research is depicted in Fig. 9. The analysis was performed using 135 keywords (used at least five times). Figure 10 demonstrates the network analysis map of keywords co-occurrence, with 135 items and eight clusters. Each item is represented by a node, and the node’s size indicates the frequency of the corresponding keyword in the field. The larger the node, the more relevant research around the keyword. The thicker the line between the two nodes, the greater the frequency of these two keywords in the subject area, and the greater the relationship between the two keywords. The keyword analysis can quickly identify the research hotspots in the field. Obvious keywords can help researchers and non-researchers quickly identify hot topics in literature research.

According to Fig. 10, the most frequently used keywords are “augmentation”, “complications”, “gluteoplasty “, “liposuction”, “gluteal augmentation”, “buttocks “, “implants”, “surgery”, “experience”, “experience “management”, “outcomes”, “efficiency”, “safety “. The different colour clusters correspond to different themes of the study. Different colour clusters are connected by lines to indicate their cross-correlation. According to Fig. 10, there are 8 related themes by colour, and “gluteoplasty” is the largest theme (Cluster 1, 49 occurrences ,TLS:229), containing 24 items, followed by “ implants” (Cluster 2, 35 occurrences ,TLS:107), “reconstruction” (Cluster 3, 18 occurrences ,TLS:31), and “efficacy” (Cluster 4, 23 occurrences ,TLS:92), “complications” (Cluster 5, 52 occurrences ,TLS:213), and “ outcomes” (Cluster 6, 24 occurrences ,TLS:74), “ augmentation” (Cluster 7, 61 occurrences , TLS:219), “ experience” (Cluster 8, 28 occurrences ,TLS:135).

**Fig. 11:**
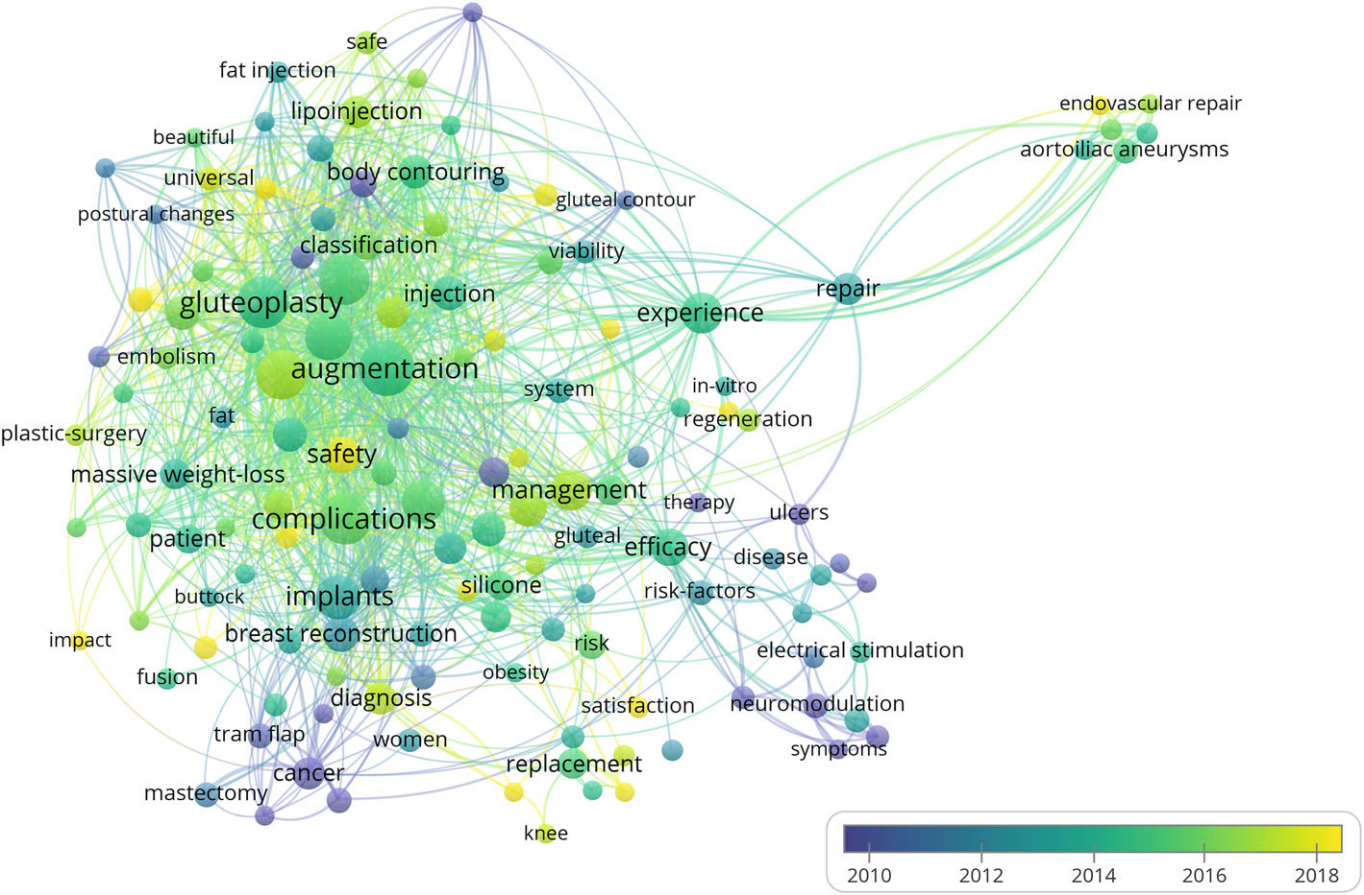
Timing analysis of keywords co-occurrence augmentation research

Figure 11 depicts the timing analysis of keywords co-occurrence of buttock augmentation research. The marks in the lower right corner of the figure indicate that we can judge the hotness of keywords in different periods based on their colour, and the nodes close to yellow indicate the hot keywords with high frequency in recent years, further indicating the development direction of research trends in this field. According to the time-series map, the research trends of buttock augmentation are focused on silicon implant, safety, satisfaction, and autologous fat grafting.

## Discussion

The buttocks are an essential component of female physical beauty and a vital component of the human body curve. Buttock augmentation has gained popularity among beauty enthusiasts as people’s living standards have improved, and the number of patients accepting buttock augmentation surgery is rapidly increasing. However, the number of publications on buttock augmentation is limited, and this study may be the first bibliometric analysis of published articles related to buttock augmentation in the existing literature.

From 1999 to 2021, the number of publications on buttock augmentation has steadily increased, and the USA has received high academic honours as a key contributor to buttock augmentation research. “Plastic and Reconstructive Surgery” is the most productive and co-cited journal in this field, indicating that it is the most informative resource in buttock augmentation research. Furthermore, “Aesthetic Plastic Surgery”, “Aesthetic Surgery Journal”, “Clinics in Plastic Surgery”, “Annals of Plastic Surgery”, “Journal of Plastic Reconstructive and Aesthetic Surgery” are the major journals in the field.

Cardenas-Camarena (Instituto Jaliscience de Cirugia Reconstructiva) is the most productive and co-cited author of gluteal augmentation publications (9 articles, 1.83%, 158 citations, TLS: 1993). The most co-cited article of the author, “Deaths Caused by Gluteal Lipoinjection: What Are We Doing Wrong?”, explored the causes of death from gluteal autologous fat injections and discovered that intramuscular fat injections in the buttocks and gluteal vascular injury were the primary causes of death [8].

Furthermore, the article “Intramuscular gluteal implants”, published in “Aesthetic Plastic Surgery”, is the most frequently co-cited reference in which the authors investigated a new space to accommodate gluteal implants, namely gluteus maximus intramuscular gluteal implants. This anatomical space within the gluteus maximus muscle can accommodate larger implants, making surgery safe and providing excellent postoperative cosmetic results [9]. This study is a great reference for the level of implant placement for buttock augmentation, which is now the main level of implant placement within the gluteus maximus muscle.

### Research Hotspots

According to the analysis of the co-cited reference network shown in Fig. 8, there are currently three research hotspots in this field: experience and technology of buttock augmentation (Cluster 1, red area), autologous fat buttock augmentation and its safety (Cluster 2, green area), and buttock aesthetics study (Cluster 3, blue area).

“Experience and technology of buttock augmentation” (Cluster 1) has the largest number of publications, with articles describing surgical techniques and sharing experiences in buttock augmentation. In the second most co-cited article, Gonzalez et al. [10] presented the “XYZ technique”, which is worth promoting since it allows for safe intragluteal muscle separation based on anatomical localization points, allowing for a perfect implant space and avoiding complications. In the fifth most co-cited article, González-Ulloa et al. [11] presented a follow-up study of patients who underwent gluteomyoplasty ten years ago, including patients with unilateral gluteus atrophy and bilateral improvement of gluteal shape, and discovered that all patients maintained good gluteal shape ten years after surgery, with the satisfaction of both patients and physicians. Furthermore, Serra et al. [12] detailed the fundamentals of gluteal anatomy and gluteal augmentation techniques to help plastic surgeons perform gluteal augmentation by identifying anatomical reference points.

“Autologous fat grafting for buttock augmentation and its safety” (Cluster 2) has shown that fat grafting to fill the buttocks, in addition to buttock augmentation with implants, is a popular procedure. Toledo et al. [13] described a thirty-year experience in Brazil with buttock augmentation by fat grafting by removing the arm, back, waist, and abdominal fat. The fat was processed, centrifuged, and injected for transplantation into the buttocks with good therapeutic effectiveness. Roberts et al. [14] used autologous microfat grafting to address the external part of the buttocks and external thighs that could not be improved by implants. This method achieved excellent outcomes. The articles with the third and fourth highest number of co-cited articles reported analysis of fat injections in the gluteal region that resulted in death, with all patients dying due to fat embolism. It also suggested that fat injections should be administered slowly, in small amounts, repeatedly, and in multiple planes, avoiding injections into the deep muscle planes of the gluteus maximus [8, 15].

The “Buttock Aesthetics Study” (Cluster 3) demonstrated that plastic surgeons and beauty enthusiasts are exploring the aesthetics of the buttocks. A full and well-rounded buttock is a defining trait and one of the secondary sexual characteristics of women. In 1993, Singh et al. [16] conducted an in-depth study on female body aesthetics and concluded that a waist-to-hip ratio of 0.7 was a widely accepted aesthetic criterion. According to body surface markings, Cuenca-Cuerra et al. [17] proposed a set of aesthetic criteria where the greater trochanter of the femur was labelled A, the most prominent point of the pubic mound was labelled B, the most prominent point of the buttocks was labelled C, and the anterior superior iliac spine was labelled D. The study also concluded that the ideal buttock form is AC:AB equals 2:1. Subsequently, Cuenca-Cuerra and Lugo-Beltran [18] concluded that a ideal buttock should have four “depressions”: the lateral depression, the short inferior gluteal fold, the superior gluteal fossa, and the V-shaped groove at the sacral triangle. Mendieta CG et al. [19] proposed a more comprehensive aesthetic assessment system and classified buttock morphology into four types: A, V, circular, and square.

### Research Trends

According to the keyword timing (Fig. 11), the research trends include the following: silicone implant, safety, satisfaction, and autologous fat grafting.

In North America, the law forbade silicone implants for buttock augmentation, and hence, only elastic implants were used. However, patients often complained of discomfort because the implants were too hard [20]. Although breast implants can also produce better buttock augmentation outcomes, their capsule membranes have shown to be weak and prone to rupture and leakage [21, 22]. Nowadays, with a full range of buttock implants, including circular and anatomical shapes, glossy and frosted surfaces, silicone rubber, and silicone gel, the development of buttock augmentation surgery has been as complete as that of breast implants [23, 24]. It is expected that further research will be conducted in support of silicone buttock implants in the future.

Safety is a priority concern for patients seeking beautiful buttocks simultaneously. Cárdenas-Camarena et al. [8] analysed and reported the causes of death due to buttock fat injections performed in Mexico and Colombia. A total of 13 of the 64 deaths related to liposuction reported in Mexico were caused by buttock fat injections, while nine deaths in Colombia were caused by fat embolism. Mofid et al. [15] also reported on the mortality of buttock fat grafting and detailed some techniques to improve the safety of the procedure. Furthermore, complications of buttock augmentation surgery are also within the scope of safety examination. Incisional dehiscence is the most common complication following buttock augmentation with implants. In the published literature, Sinno et al. [25] collected 2375 cases of buttock augmentation, with a 9.6% incidence of postoperative incisional dehiscence. Another study has shown incidences of incisional dehiscence up to 30% after buttock augmentation with implants [26]. Cárdenas-Camarena et al. [27] showed a 4.4% probability of seroma after buttock augmentation. The rate of infection after buttock augmentation with implants was 3.2% [28]. The overall incidence of implant displacement, ectopic position, rotation, or buttock asymmetry was about 2.3% [24]. The incidence of contracture of the buttock implant envelope was 3.5% [29]. Condé-Green et al. [30] performed a meta-analysis of postoperative complications in 4084 patients who underwent autologous lipofilling of the buttocks. The incidence of each complication was seroma (2.40%), rash (1.30%), pain (0.76%), morphological abnormalities (0.64%), and fat necrosis (0.56%), in that order.

Satisfaction, the ultimate criterion for evaluating the outcome of buttock augmentation, is closely related to safety, complications, and postoperative experience of the procedure. With the increasing number of buttock augmentation procedures, there is an urgent need to explore a standardized method for measuring patient satisfaction.

First reported in 1990 by Chajchir without a detailed description of the case, autologous fat grafting for buttock augmentation is an evolving field [31]. Subsequently, the use of autologous fat grafting for buttock augmentation has been reported successively [32, 33]. Currently, for patients with lateral thigh depressions that cannot be covered by implants, autologous fat grafting of the buttocks has become the predominant buttock augmentation approach. Its most significant advantage is that the entire buttock can be finely shaped by liposuction and fat injection, with few complications and minimal surgical scarring [34]. According to the American Society for Aesthetic Plastic Surgery 2018 data, 94% of buttock augmentation procedures are autologous fat-filled buttock augmentation, and only 6% are implants [35].

### Strengths and Limitations

The study assessed the current status of buttock augmentation research, research hotspots, and research trends through bibliometric analysis. However, there are some limitations in the study; the analysis of this study retrieved content only from the English language publications in the WOSCC database, causing language bias; this study has omitted publications that are not included in the WOSCC database. Furthermore, with the increased number of publications related to buttock augmentation in recent years, some newly published high-quality publications have low citation frequency and are easily missed.

## Conclusion

The USA is the dominant contributor to buttock augmentation research, followed by Germany, Brazil, and the UK. The New York University (USA) has the most publications on buttock augmentation. Cardenas-Camarena (Instituto Jaliscience de Cirugia Reconstructiva) is the most productive and most co-cited author on buttock augmentation. “Plastic and Reconstructive Surgery” is the most productive and most co-cited journal in the field. Research hotspots include experience and technology of buttock augmentation, autologous fat buttock augmentation and its safety, and buttock aesthetics study. It is believed that more publications will be published in the future, and the research trends will revolve around silicone implants, safety, satisfaction, and autologous fat grafting.
